# Tim-3 downregulation by *Toxoplasma gondii* infection contributes to decidual dendritic cell dysfunction

**DOI:** 10.1186/s13071-022-05506-1

**Published:** 2022-10-27

**Authors:** Hongbing Xie, Zhidan Li, Guangmei Zheng, Chunyan Yang, Xianbing Liu, Xiaoyan Xu, Yushan Ren, Chao Wang, Xuemei Hu

**Affiliations:** 1 Department of Immunology, Binzhou Medical University, Yantai, 264003 Shandong People’s Republic of China; 2 Department of Oral Biology, Binzhou Medical University, Yantai, 264003 Shandong People’s Republic of China

**Keywords:** *Toxoplasma gondii*, Maternal–fetal tolerance, Decidual dendritic cells, T cell immunoglobulin and mucin domain-containing protein 3, Adverse pregnancy outcome

## Abstract

**Background:**

Women in early pregnancy infected by *Toxoplasma gondii* may have severe adverse pregnancy outcomes, such as spontaneous abortion and fetal malformation. The inhibitory molecule T cell immunoglobulin and mucin domain 3 (Tim-3) is highly expressed on decidual dendritic cells (dDCs) and plays an important role in maintaining immune tolerance. However, whether *T. gondii* infection can cause dDC dysfunction by influencing the expression of Tim-3 and further participate in adverse pregnancy outcomes is still unclear.

**Methods:**

An abnormal pregnancy model in Tim-3-deficient mice and primary human dDCs treated with Tim-3 neutralizing antibodies were used to examine the effect of Tim-3 expression on dDC dysfunction after *T. gondii* infection.

**Results:**

Following *T. gondii* infection, the expression of Tim-3 on dDCs was downregulated, those of the pro-inflammatory functional molecules CD80, CD86, MHC-II, tumor necrosis factor-α (TNF-α), and interleukin-12 (IL-12) were increased, while those of the tolerant molecules indoleamine 2,3-dioxygenase (IDO) and interleukin-10 (IL-10) were significantly reduced. Tim-3 downregulation by *T. gondii* infection was closely associated with an increase in proinflammatory molecules and a decrease in tolerant molecules, which further resulted in dDC dysfunction. Moreover, the changes in Tim-3 induced by *T. gondii* infection further reduced the secretion of the cytokine IL-10 via the SRC-signal transducer and activator of transcription 3 (STAT3) pathway, which ultimately contributed to abnormal pregnancy outcomes.

**Conclusions:**

*Toxoplasma gondii* infection can significantly downregulate the expression of Tim-3 and cause the aberrant expression of functional molecules in dDCs. This leads to dDC dysfunction, which can ultimately contribute to abnormal pregnancy outcomes. Further, the expression of the anti-inflammatory molecule IL-10 was significantly decreased by Tim-3 downregulation, which was mediated by the SRC-STAT3 signaling pathway in dDCs after *T. gondii* infection.

**Supplementary Information:**

The online version contains supplementary material available at 10.1186/s13071-022-05506-1.

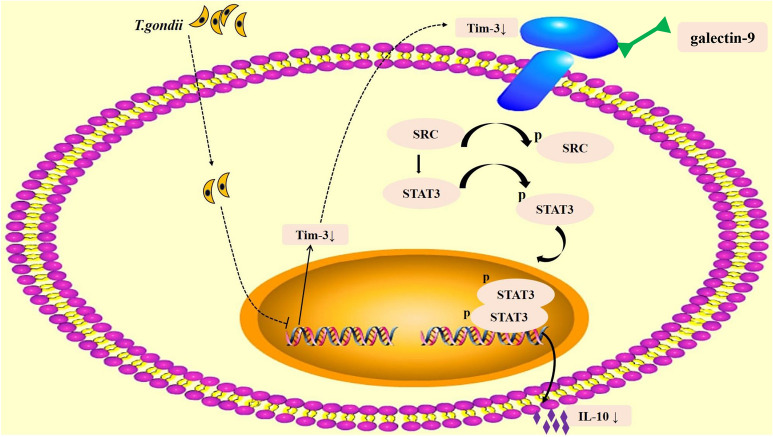

## Background

*Toxoplasma gondii* is an obligate intracellular protozoan parasite that can infect almost all warm-blooded vertebrates [1, 2]. Infection in early pregnancy can spread vertically to the fetus, leading to severe abnormal pregnancy outcomes, such as miscarriage, intellectual disability and congenital malformation [3, 4]. The immune microenvironment at the maternal–fetal interface plays an important role in maintaining normal pregnancy [5]. The decidual immune system consists of a variety of maternal immune cells, such as natural killer (NK) cells, macrophages (Mφs), and dendritic cells (DCs), which play important roles in maintaining maternal–fetal tolerance [6–8]. Dysfunction of these immune cells due to the effects of various external stimuli can lead to many types of adverse pregnancy outcomes [8–10]. Decidual DCs (dDCs) account for approximately 1–7% of mononuclear cells, and are specifically equipped to control immunity, trigger immune response and maintain tolerance, preventing the rejection of the conceptus by the maternal immune system [11, 12]. Mammalian DCs are divided into at least two subsets, described as myeloid DCs and plasmacytoid DCs [13]. It was reported that dDC dysfunction was closely related to the occurrence of spontaneous abortion [14, 15]. The tolerant role of dDCs at the maternal–fetal interface is largely dependent on the expression of inhibitory molecules, such as T cell immunoglobulin domain and mucin domain 3 (Tim-3), leukocyte immunoglobulin-like receptor B4 (LILRB4) and B7 homolog 4 (B7-H4) [16–18]. Previous studies in our group showed that *T. gondii* infection could downregulate the expression of these regulatory molecules on dDCs or dMφs and further contribute to adverse pregnancy outcomes [19–21]. However, how the inhibitory molecule T cell immunoglobulin and mucin domain 3 (Tim-3) on dDCs is involved in *T. gondii* infection-mediated abnormal pregnancy has not, to the best of our knowledge, been previously reported.

Tim-3 was initially identified as a Th1-specific cell surface molecule involved in the transduction of apoptotic signals, which could inhibit the Th1 response via galectin-9 activation [22]. Recently, several studies have reported that Tim-3 is also highly expressed on several decidual immune cells, such as DCs, monocyte-Mφs, regulatory T cells, and NK cells, maintaining maternal–fetal tolerance by modulating the functions of these immune cells [23–25]. Another study showed that pregnant mice that were treated with Tim-3-blocking antibodies exhibited obvious fetal loss with altered Th1/2 cytokine profiles [26]. Our previous studies showed that *T. gondii* infection could downregulate the expression of Tim-3 on dMφs and dNK cells, and further resulted in cell dysfunction that contributed to adverse pregnancy outcomes [19, 20]. However, whether *T. gondii* infection can affect the expression level of Tim-3 on dDCs and lead to dDC dysfunction remains to be confirmed. There is evidence that blocking the Tim-3 pathway in decidual immune cells lowers the production of interleukin-10 (IL-10) [16]. Another study suggested that Tim-3 negatively regulated interleukin-12 (IL-12) expression in monocytes during hepatitis C virus infection [27]. A study also showed that Tim-3^+^ dNK cells secreted lower levels of tumor necrosis factor-α (TNF-α) [28]. However, whether the changes in these functional molecules are caused by the downregulation of Tim-3 on dDCs after *T. gondii* infection is still unclear. There is evidence that healthy pregnancy is associated with higher levels of IL-10, while pathologic pregnancies are associated with lower levels of IL-10, suggesting that IL-10 plays an important role in maintaining maternal–fetal tolerance [29]. Tim-3 can inhibit the activation of DCs through Bruton’s tyrosine kinase -SRC signaling pathway [30]. Alcohol can activate SRC kinase, and activated SRC kinase directly activates signal transducer and activator of transcription 3 (STAT3) at the Tyr705 residue [31]. Phosphorylated STAT3 (p-STAT3) dimerizes, translocates to the nucleus and binds to the promoter residues of IL-10 to regulate the expression of IL-10 [32]. Hence, whether *T. gondii* infection can regulate the expression of IL-10 by Tim-3 downregulation-mediated SRC-STAT3 signaling pathway and further result in dDCs dysfunction remains unclear. In the present study, pregnant Tim-3-deficient (Tim-3^−/−^) female mice and primary human dDCs treated with Tim-3 neutralizing antibodies were used to investigate the effect of Tim-3 on dDC dysfunction during *T. gondii* infection and clarify the molecular mechanism of abnormal pregnancy outcomes.

## Methods

### Animal models

C57BL/6 mice [wild-type (WT) mice; 6- to 8-week-old females; 8- to 10-week-old males] were purchased from Pengyue Laboratory Animal Technology (Jinan, China). Tim-3^−/−^ mice were purchased from Bioray Laboratories (Shanghai, China). All of the mice were raised under specific pathogen-free conditions at 22–26 ℃, 50–60% humidity and a 12-h/12-h light/dark cycle, and were supplied with sufficient sterile water and food. Females and males were housed overnight in one cage at a ratio of 2:1. Female mice with vaginal plugs on gestational day (GD) 0 were segregated and randomized into three groups: uninfected, infected, and Tim-3^−/−^ infected. On GD 8, mice in the infected group and Tim-3^−/−^ infected group were intraperitoneally injected with 400 tachyzoites resuspended in 200 μl phosphate buffered saline (PBS); the uninfected mice were injected with the same volume of PBS into the abdominal cavity. All procedures performed on animals in this study were conducted according to the ethical standards of the Ethics Committee and Institutional Animal Experimental Ethics Committee of Binzhou Medical University (permit no. 2017-009-09). All of the animal experiments were performed on animals under sodium pentobarbital anesthesia to minimize their suffering, all reagents used in present study were listed in the Additional file 1 (Table S1).

### Genotyping Tim-3^−/−^ mice

Genomic DNA of Tim-3^−/−^ mice was extracted from a 1-mm section of tail tissue using a tissue DNA extraction kit (Generay, China). The primers for Tim-3 were as follows: sense, 5′-GGCTGGCTCAAACTCACTACA-3′; anti-sense, 5′-CGGACAATGATAACATGGAAA-3′. The complementary DNA product was amplified by polymerase chain reaction in accordance with the manufacturer’s instructions (Generay). Initially, the product was denatured at 95 ℃ for 3 min, then the PCR performed with 30 cycles of denaturation of 30 s at 95 ℃, annealing for 30 s at 56 ℃, extension for 60 s at 72 ℃, followed by a final extension for 8 min at 72 ℃ and maintenance temperature of 16 ℃. Then, the PCR products were sequenced (MDbio, China), and the homozygotes, heterozygotes and WT mice distinguished by matching the DNA sequence to the WT sequence. The homozygotic Tim-3^−/−^ mice obtained were then bred.

### Scanning electron microscopy

The pregnant female mice in the three groups were euthanized on GD 14, and the fetuses harvested by separating them from the uteruses. The fetuses were washed five times with 0.1 mol PBS and then fixed in 2.5% buffered glutaraldehyde for 1 week at 4 ℃. The fetuses were then dried in specimen holders by the critical point technique (Quorum K850) and coated with gold particles by an ion sputter coater (Quorum Q150RS). Finally, all the specimens were observed under a scanning electron microscope (ZEISS EVO LS15) operated at 10 kV, and images obtained with SmartSEM user-interface software.

### Obtainment and reproduction of *T. gondii* tachyzoites

*T. gondii* tachyzoites were cultured in Hep-2 cells, which were grown in minimum essential medium (HyClone, USA), 5% fetal bovine serum (Gibco, USA), and 100 IU/ml penicillin/streptomycin (Solarbio, China). To acquire *T. gondii* tachyzoites, the cells and culture media were collected and the cells were removed by centrifugation at 800 r.p.m. (433 × *g*) for 5 min; the tachyzoites were then purified by centrifugation at 4000 r.p.m. (2810 ×* g*) for 7 min. The acquired *T. gondii* tachyzoites were resuspended in minimum essential medium and counted using a Neubauer chamber. Consumable experimental instruments and liquids contaminated by tachyzoites were disinfected and autoclaved immediately.

### Mouse cell preparation

On GD 14, the mouse uterus and placenta were carefully separated and washed two to three times with cold PBS. The tissues were cut into 1- to 3-mm fragments with ophthalmic scissors. Then, 1 mg/ml collagenase IV (Biofroxx, Germany) and 0.2 mg/ml deoxyribonuclease (DNase; Sigma–Aldrich, St. Louis, Mo) were added, and the samples digested in a biochemical incubator at 37 ℃ for 45 min. The digested tissue was then filtered through 48-μm sterile nets and washed twice in cold PBS. Finally, the cells were collected and resuspended in cold PBS. Mononuclear cells were obtained by aspirating the white membrane layer in the mouse lymphocyte isolation medium (TBD Science, China) after Ficoll density gradient centrifugation, and were used for flow cytometry.

### Collection of human clinical samples

The human clinical decidual tissue used in this study was provided by the Department of Obstetrics and Gynecology of Yantai Affiliated Hospital of Binzhou Medical University and the Yantai Zhifu District Material and Child Health Hospital. The aborted tissues of healthy pregnant women who underwent voluntary abortion during the first trimester of pregnancy (6–8 weeks of gestation) were collected under aseptic conditions. The tissue was immediately flushed five to eight times with sterile normal saline solution, and decidual tissues were collected in RPMI 1640 (HyClone, USA) supplemented 100 IU/ml penicillin/streptomycin (Solarbio, China). The sample collection procedure was approved by the Ethics Committee of Binzhou Medical University (approval no. 2017-016-01). A total of 50 pregnant women aged 25–45 years old were enrolled in this study. The patients voluntarily underwent abortions, and signed informed consent forms. All the pregnancies were diagnosed by a professional obstetrician and gynecologist as early normal gestation without any evidence of a threatened abortion or any complications, or any ongoing medication of the pregnant women.

### Isolation and purification of human dDCs

The decidual tissue was washed five to six times with cold PBS and then cut into 1- to 3-mm fragments with ophthalmic scissors. Then, 1 mg/ml collagenase IV (Biofroxx, Germany) and 0.2 mg/ml DNase (Sigma‒Aldrich, St. Louis, USA) were added to digest the tissue in a biochemical incubator at 37 ℃ for 45 min. The digested tissue was filtered through 48-μm sterile nets and washed twice in cold PBS. Finally, the cells were collected and resuspended in cold PBS. After Ficoll density gradient centrifugation at 2000 r.p.m. for 20 min, mononuclear cells were obtained by aspirating the white membrane layer in human lymphocyte isolation medium (TBD Science) in accordance with the manufacturer’s instructions. Untouched and highly purified myeloid DCs were isolated from fresh decidual tissue mononuclear cells with purities of up to 90% (Stemcell, Canada). The purified human dDCs were used for western blotting analysis.

### Flow cytometry

The mouse antibodies, FITC-conjugated anti-CD11c, PerCP/Cy5.5-conjugated anti-CD8α, and PE-conjugated anti-IL-10/IL-12 were purchased from BD (USA). BV421-conjugated anti-Tim-3 and PE-conjugated anti-CD80, anti-CD86, and anti-MHC-II were purchased from Biolegend (USA). APC-conjugated anti-indoleamine 2,3-dioxygenase (anti-IDO) and PE-conjugated anti-TNF-α were purchased from eBioscience (USA). The following fluorochrome-conjugated monoclonal antibodies (mAbs) were used for the human samples: PE/CY7-conjugated anti-CD86 and IL-10 (purchased from Biolegend). FITC-conjugated anti-HLA-DR, PerCP/Cy5.5-conjugated anti-lineage, and PE-conjugated anti-CD80 and IDO were purchased from eBioscience.

The mouse or human decidual mononuclear cells were first incubated with the corresponding membrane mAbs against the cell surface molecules, including Tim-3, CD80, CD86, and MHC-II (mouse) or HLA-DR (human) at 4 ℃ in the dark for 30 min, and washed once. Then, the cells were fixed and permeabilized using an eBioscience kit, in accordance with the manufacturer’s instructions, and washed twice. Intracellular antibodies (anti-IDO, anti-IL-10, anti-IL-12, anti-TNF-α) were added to the cells, which were then incubated at 4 ℃ for 40 min in the dark. To examine cytokines, the cells were first treated with a leukocyte activation cocktail (eBioscience) for 6 h. They were then incubated with the corresponding membrane mAbs, fixed and permeabilized, and subsequently incubated with intracellular antibodies (anti-IL-10, anti-IL-12, anti-TNF-α) as described above. Flow cytometry was performed using a FACSCanto II flow cytometry system (Becton Dickinson, Franklin Lakes, NJ) with FlowJo v.10.3 software (FlowJo, Ashland, OR).

### Western blotting analysis

The human dDCs were treated with anti-Tim-3 neutralized antibodies, dasatinib (a SRC inhibitor) or Stattic (a STAT3 inhibitor) for 2 h, then infected with *T. gondii* for 24 h. Then, a total of 1 × 10^7^ cells of each group were collected and lysed with the pre-cooled lysis buffer, with 10 µl of the serine protease inhibitor phenylmethylsulfonyl fluoride (PMSF) per milliter of buffer. After incubation for 45 min on ice, the cell lysates were centrifuged at 12,000 × *g* at 4 ℃, the supernatant collected, quantified by BCA protein assay kits and mixed with 5× sodium dodecyl sulfate–polyacrylamide gel electrophoresis loading buffer, following by boiling for 5 min. Then, equal amounts of protein were separated by 12% sodium dodecyl sulfate–polyacrylamide gel electrophoresis. The proteins were transferred to polyvinylidene fluoride membranes (Millipore, Germany). The membranes were then blocked at room temperature for 2 h in 5% skim milk in Tris buffered saline–Tween buffer. The membranes were incubated on a shaker overnight at 4 ℃ with rabbit anti-human Tim-3 (1:2000 dilution; Bioss, China), rabbit anti-human STAT3/SRC (1:2000 dilution; Proteintech, China), IL-10/IL-12 (Abcam, UK), and rabbit anti-human p-STAT3/p-SRC (1:2000 dilution; CST, USA). Then the membranes were washed 5 times in Tris buffered saline–Tween and incubated with horseradish peroxidase conjugated goat anti-rabbit IgG secondary antibody (1:2000 dilution; Protech, USA) at room temperature for 1 h. After washing five times for 20 min, the proteins on the membranes were visualized by an enhanced chemiluminescence kit (Roche, Switzerland) at appropriate exposure times. Glyceraldehyde-3-phosphate dehydrogenase was analyzed using rabbit anti-glyceraldehyde-3-phosphate dehydrogenase polyclonal antibody (Protech) as a loading control.

### Histopathology

Placentas were removed from pregnant mice on GD 14, washed five times with PBS and fixed in 4% paraformaldehyde for 1 week. The tissues were removed and washed with tap water for 4 h, followed by gradient alcohol dehydration. The placental tissue was embedded in paraffin and cut into 5-µm slices. The thin slices were stained with hematoxylin and eosin (H&E; Shanghai Novland, China) dye according to the instructions in the manufacturer’s manual. Images were observed and recorded under a microscope at 20× magnification.

### Statistical analysis

The data are presented as the means ± SDs. All the statistical analyses were performed using the GraphPad Prism 8 statistical software package. Unpaired *t*-tests or paired *t*-tests were used to identify differences.

## Results

### Tim-3 expression on dDCs was decreased after* T. gondii* infection

**Fig. 1:**
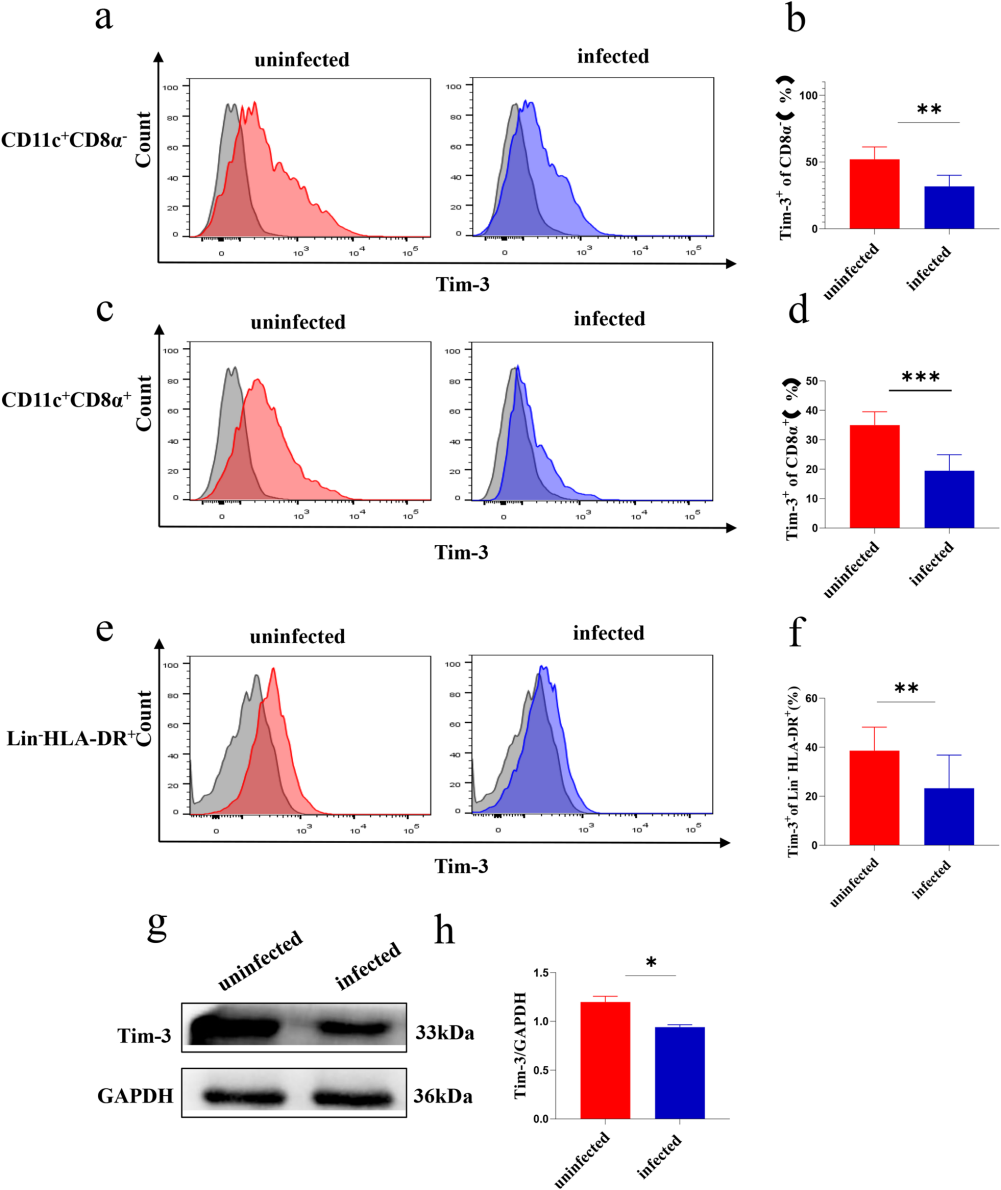
**a**–**h** Expression of T cell immunoglobulin and mucin domain 3 (Tim-3) on human and murine decidual dendritic cells (dDCs) after *Toxoplasma gondii* infection. **a** The expression of Tim-3 on myeloid DCs (CD11c^+^CD8α^−^) from uninfected and infected mice was examined by flow cytometry. **b** The changes in Tim-3 levels in the uninfected and infected groups were statistically analyzed. **c** The expression of Tim-3 on the surface of lymphatic DCs (CD11c^+^CD8α^+^) from uninfected and infected mice was examined by flow cytometry. **d** The changes in Tim-3 levels between the uninfected and infected groups were statistically analyzed. **e** The expression of Tim-3 on myeloid DCs (lineage^−^ HLA–DR^+^) from uninfected and infected human decidua was examined by flow cytometry. **f** The changes in Tim-3 expression in the uninfected and infected groups were statistically analyzed. **g** The expression of Tim-3 on human myeloid DCs from the uninfected and infected groups was examined by western blotting. **h** The changes in Tim-3 expression between the uninfected and infected groups were statistically analyzed. The data shown are the means ± SD. At least six pregnant mice in each group were assayed individually, and differences in the results were identified by unpaired *t*-test: uninfected vs. infected group. At least six samples of human decidua in each group were assayed individually by flow cytometry, and at least three samples of human decidua in each group were assayed individually by western blotting. Differences in the results were identified by paired *t*-test. * P < 0.05, ** P < 0.01, *** P < 0.001

Firstly, the expression of Tim-3 on dDC subsets was examined between uninfected and *T. gondii*-infected mice by flow cytometry and western blot. The Tim-3 expression levels in murine myeloid and lymphatic dDCs were decreased [CD11c^+^CD8α^−^, *t*-test, *t*(10) = 3.959, *P* = 0.0027; CD11c^+^CD8α^+^, *t*-test, *t*(10) = 5.335, *P* = 0.0003] after *T. gondii* infection (Fig. 1a–d). Similarly, the Tim-3 expression levels on human dDCs showed a significant decrease after *T. gondii* infection according to flow cytometry [Lin^−^HLA-DR^+^, *t*-test, *t*(5) = 5.927, *P* = 0.0019] and western blotting [*t*-test, *t*(2) = 5.472, *P* = 0.0318] (Fig. 1e–h; Additional file 2).

### The adverse pregnancy outcome model was successfully established in infected and pregnant Tim-3^−/−^ mice

**Fig. 2:**
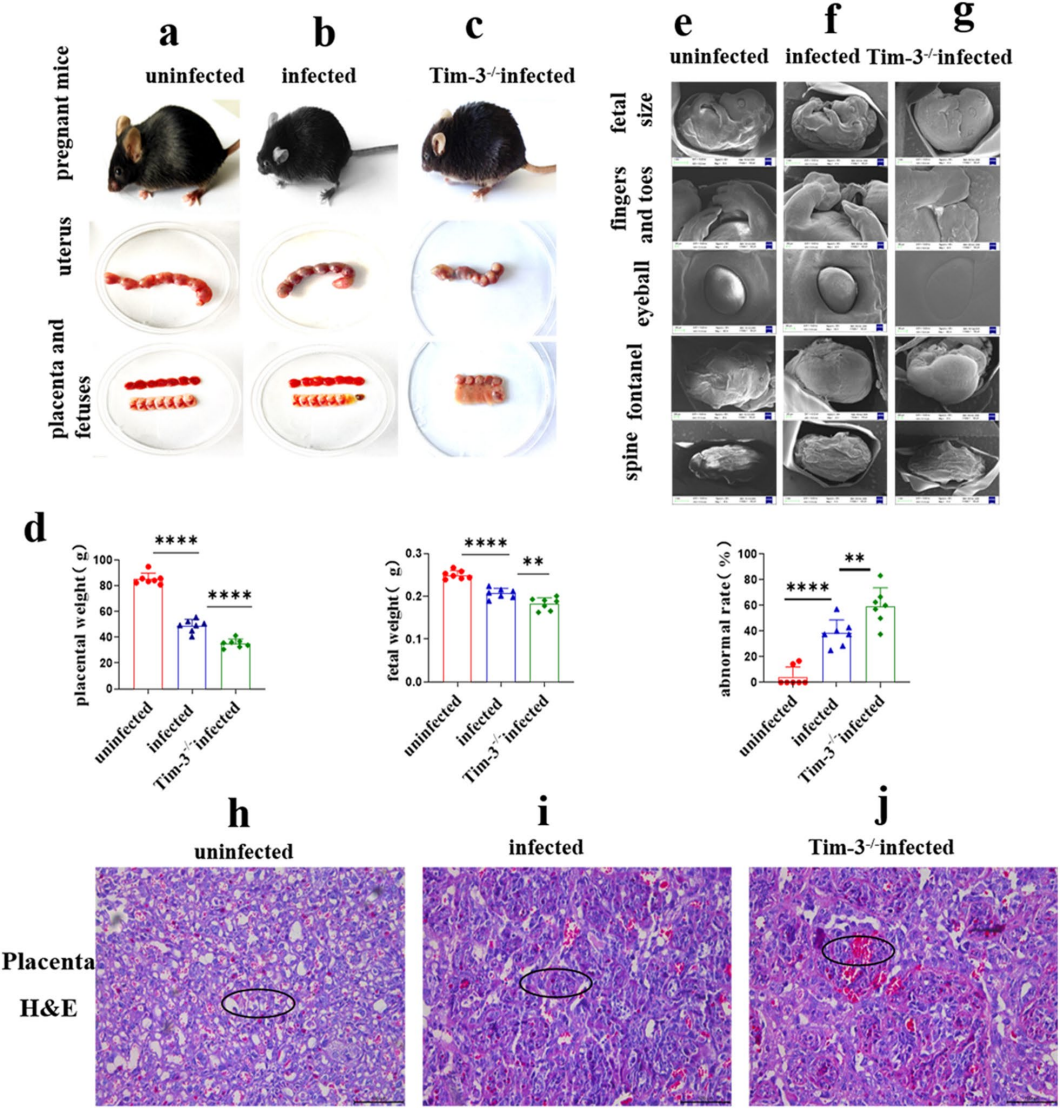
**a** Impact of Tim-3 on adverse pregnancy outcomes caused by *Toxoplasma gondii* infection in mice. **a** The mental states of uninfected pregnant mice were good, and the fetal mice developed well. **b** Pregnant mice infected with *T. gondii* showed listlessness, and the placentas and fetuses exhibited inflammatory congestion. **c** Tim-3-deficient (*Tim-3*^−/−^)-infected pregnant mice had worse mental states, more stillbirths, and their fetuses were absorptive or stunted. **d** The weights of the placentas and fetuses and the abnormal rates in uninfected, infected and infected Tim-3^−/−^ pregnant mice were statistically analyzed. **e**–**g** The developmental state of fetuses was observed by scanning electron microscopy. **h**–**j** Placentas from uninfected, infected, and infected and pregnant Tim-3^−/−^ mice were stained with hematoxylin and eosin (*H*&*E*) and showed severe bleeding, erythrocyte aggregation and cellular necrosis (black circle). At least three pregnant mice in each group were assayed individually, and differences in the results identified by unpaired *t*-test. * P < 0.05, ** P < 0.01, *** P < 0.001, **** P < 0.0001

The pregnant mice were infected with 400 T*. gondii* tachyzoites on GD 8 and the pregnancy outcomes were observed on GD 14. Placentas and uteruses were dissected carefully from uninfected, infected and infected and pregnant Tim-3^−/−^ mice. Infected and pregnant Tim-3^−/−^ mice had worse adverse pregnancy outcomes, including lower placental weight [*t*-test, *t*(12) = 6.107, *P* < 0.0001] and fetal weight [*t*-test, *t*(12) = 3.494, *P* = 0.0044] and higher ratios of abnormal fetuses [*t*-test, *t*(12) = 3.189, *P* = 0.0078] than infected pregnant mice (Fig. 2a–d). H&E staining in infected and pregnant Tim-3^−/−^ mice showed severe bleeding, erythrocyte aggregation and cellular necrosis compared to infected pregnant mice (Fig. 2h–j). Scanning electron microscopy showed that the fetal physique was decreased, the toe web was not developed, the development of the eyeball and spine was not perfect, and the fontanelle was closed prematurely (Fig. 2e–g).

### Tim-3 downregulation after *T. gondii* infection could change the expression of functional membrane molecules on dDCs

**Fig. 3:**
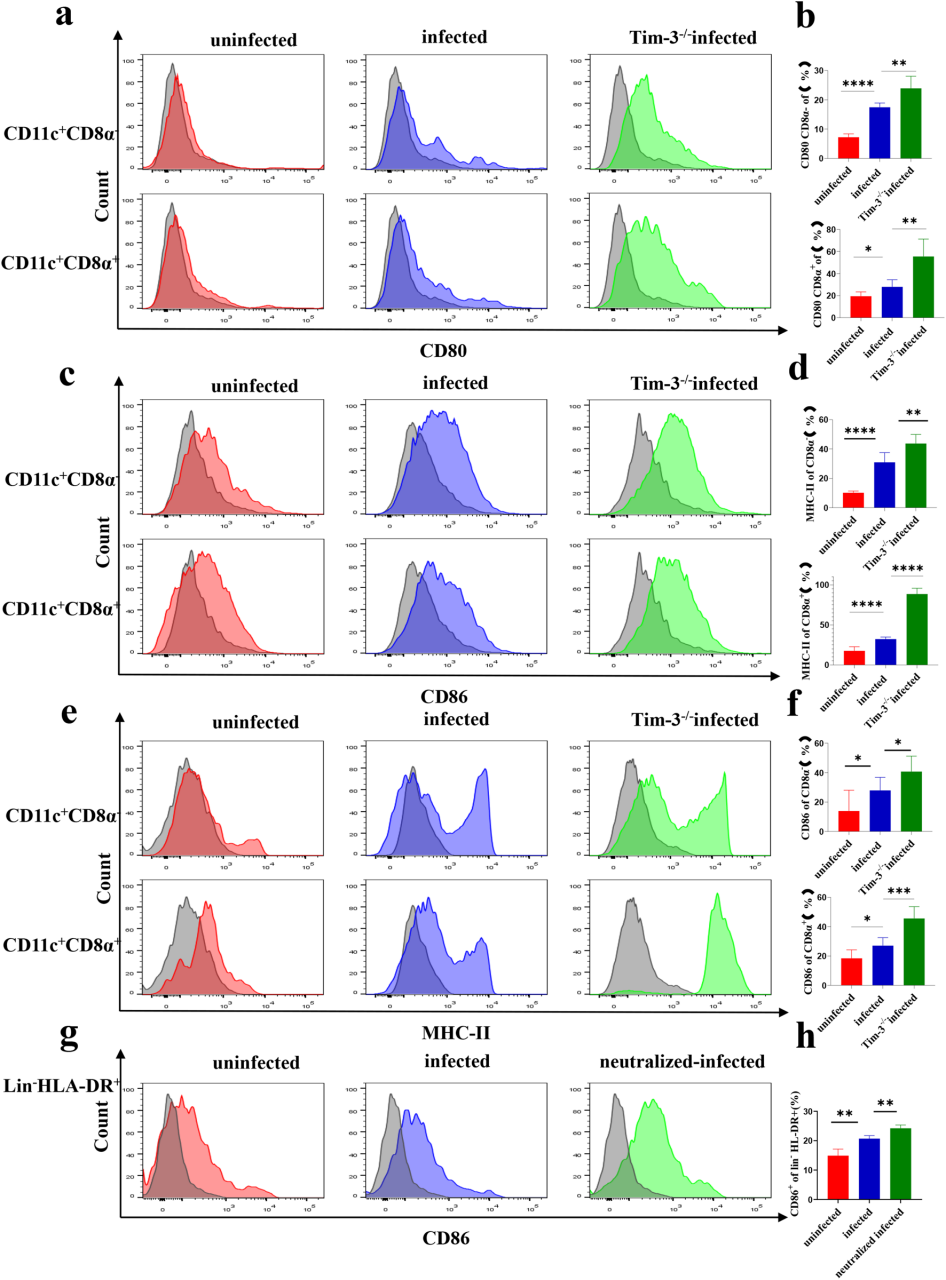
a–h Downregulation of Tim-3 on human and murine dDCs by *Toxoplasma gondii* infection is related to the expression levels of the co-stimulatory molecules CD80, CD86, and MHC-II. The expression of CD80 (**a**), CD86 (**c**) and MHC-II (**e**) on myeloid dDCs and lymphoid dDCs from uninfected, infected, and infected and Tim-3^−/−^ mice was examined by flow cytometry. Changes in the expression of CD80 (**b**), CD86 (**d**) and MHC-II (**f**) on myeloid dDCs and lymphoid dDCs from uninfected, infected, and infected and Tim-3^−/−^ mice were statistically analyzed. **g** The expression of CD86 on human dDCs from the uninfected, infected and Tim-3-neutralized infected groups was examined by flow cytometry. **h** Changes in the expression of CD86 on human dDCs from the uninfected, infected and Tim-3-neutralized plus infected groups were statistically analyzed. The data shown are the means ± SD. At least six samples of human decidua in each group were assayed individually by flow cytometry, and differences in the results identified by a paired *t*-test. At least six pregnant mice in each group were assayed individually, and differences in the results identified by unpaired *t*-test: uninfected vs. infected group. * P < 0.05, ** P < 0.01, *** P < 0.001, **** < 0.0001

To examine the effect of Tim-3 downregulation after *T. gondii* infection on the functional membrane molecules of dDCs, infected pregnant WT mice and infected and pregnant Tim-3^−/−^ mice were used to examine the functional molecules CD80, CD86, and MHC-II levels on murine dDCs by flow cytometry in vivo. CD80, CD86, MHC-II levels in infected Tim-3^−/−^ mice were higher than in infected WT mice [CD80 including CD11c^+^CD8α^−^, *t*-test, *t*(10) = 3.609, *P* = 0.0048 and CD11c^+^CD8α^+^, *t*-test, *t*(10) = 3.935, *P* = 0.0028; CD86 including CD11c^+^CD8α^−^, *t*-test, *t*(10) = 3.458, *P* = 0.0061 and CD11c^+^CD8α^+^, *t*-test, *t*(10) = 17.86, *P* < 0.0001; MHC-II including CD11c^+^CD8α^−^, *t*-test, *t*(12) = 2.463, *P* = 0.0299 and CD11c^+^CD8α^+^, *t*-test, *t*(10) = 2.662, *P* = 0.0238] (Fig. 3a–f). Tim-3 neutralizing antibodies were added for in vitro culture of human dDCs that were infected with *T. gondii*. CD86 levels of human dDCs were examined by flow cytometry. CD86 expression in Tim-3-neutralized and infected primary human dDCs was higher than that in infected dDCs alone [Lin^−^HLA-DR^+^, *t*-test, *t*(5) = 6.811, *P* = 0.0010] (Fig. 3g, h). These results indicated that Tim-3 downregulation on dDCs by *T. gondii* infection increased CD80, CD86, and MHC-II expression.

### Tim-3 downregulation after *T. gondii* infection decreased IDO and IL-10 expression in dDCs

**Fig. 4:**
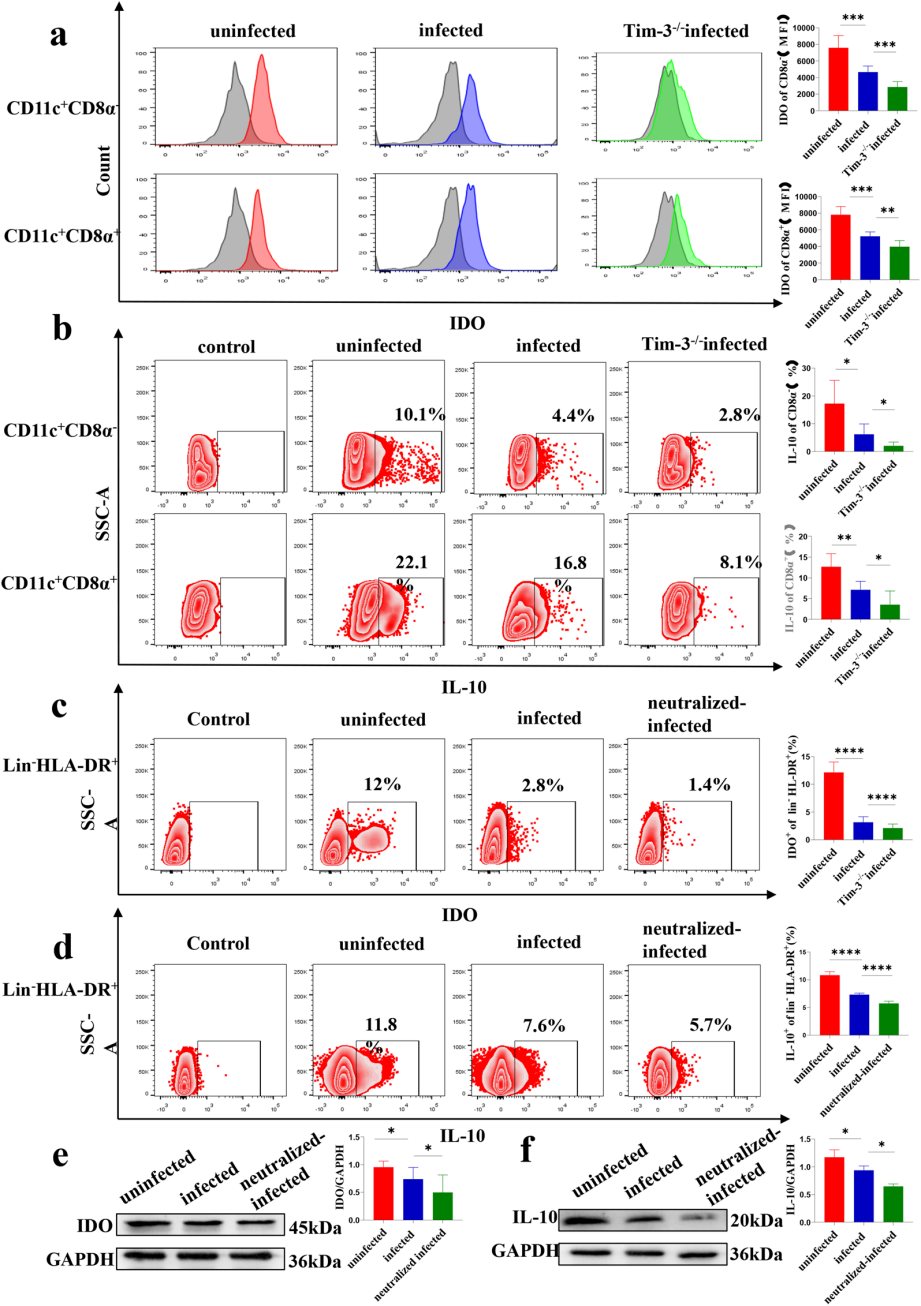
a–f Tim-3 downregulation on human and murine dDCs by *Toxoplasma gondii* infection is associated with the expression of indoleamine 2,3-dioxygenase (*IDO*) and interleukin-10 (*IL-10*). The expression of IDO (**a**) and IL-10 (**b**) on myeloid and lymphoid dDCs from uninfected, infected, and infected and Tim-3^−/−^ mice was examined by flow cytometry and the results statistically analyzed. The expression of IDO (**c**) and IL-10 (**d**) on human myeloid dDCs from the uninfected, infected and Tim-3-neutralized plus infected groups was examined by flow cytometry and statistically analyzed. The levels of IDO (**e**) and IL-10 (**f**) in human dDCs from the uninfected, infected and Tim-3-neutralized infected groups were examined by western blotting. The data shown are the means ± SD. At least six samples of human decidua in each group were assayed individually by flow cytometry, and at least three samples of human decidua in each group were assayed individually by western blot. Differences in the results were identified by paired *t*-test. At least six pregnant mice in each group were assayed individually, and differences in the results identified by unpaired *t*-test: uninfected vs. infected group. * P < 0.05, ** P < 0.01, *** P < 0.001, **** P < 0.0001

IDO and IL-10 levels were analyzed by flow cytometry to determine the effect of Tim-3 downregulation after *T. gondii* infection on IDO expression. The expression of IDO in infected Tim-3^−/−^ mouse dDCs was decreased compared with that in infected WT mice [CD11c^+^CD8α^−^, *t*-test, *t*(12) = 4.862 *P* = 0.0004; CD11c^+^CD8α^+^, *t*-test, *t*(10) = 3.359, *P* = 0.0073] (Fig. 4a). IL-10 expression was lower in infected Tim-3^−/−^ mouse dDCs than in infected WT mice [CD11c^+^CD8α^−^, *t*-test, *t*(10) = 2.583 *P* = 0.0273; CD11c^+^CD8α^+^, *t*-test, *t*(10) = 3.661, *P* = 0.0044] (Fig. 4b). Similar to the results for murine dDCs, IDO expression and IL-10 expression in the Tim-3-neutralization infection group of human dDCs were downregulated compared with those in the infected human dDCs groups [Lin^−^HLA-DR^+^, *t*-test, *t*(6) = 6.213, *P* = 0.0008], [Lin^−^HLA-DR^+^, *t*-test, *t*(5) = 15.17, *P* < 0.0001] (Fig. 4c–f). These results suggested that Tim-3 downregulation after *T. gondii* infection decreased the synthesis of IDO and IL-10.

### Tim-3 downregulation after *T. gondii* infection increased the expression of IL-12 and TNF-α in dDCs

**Fig. 5:**
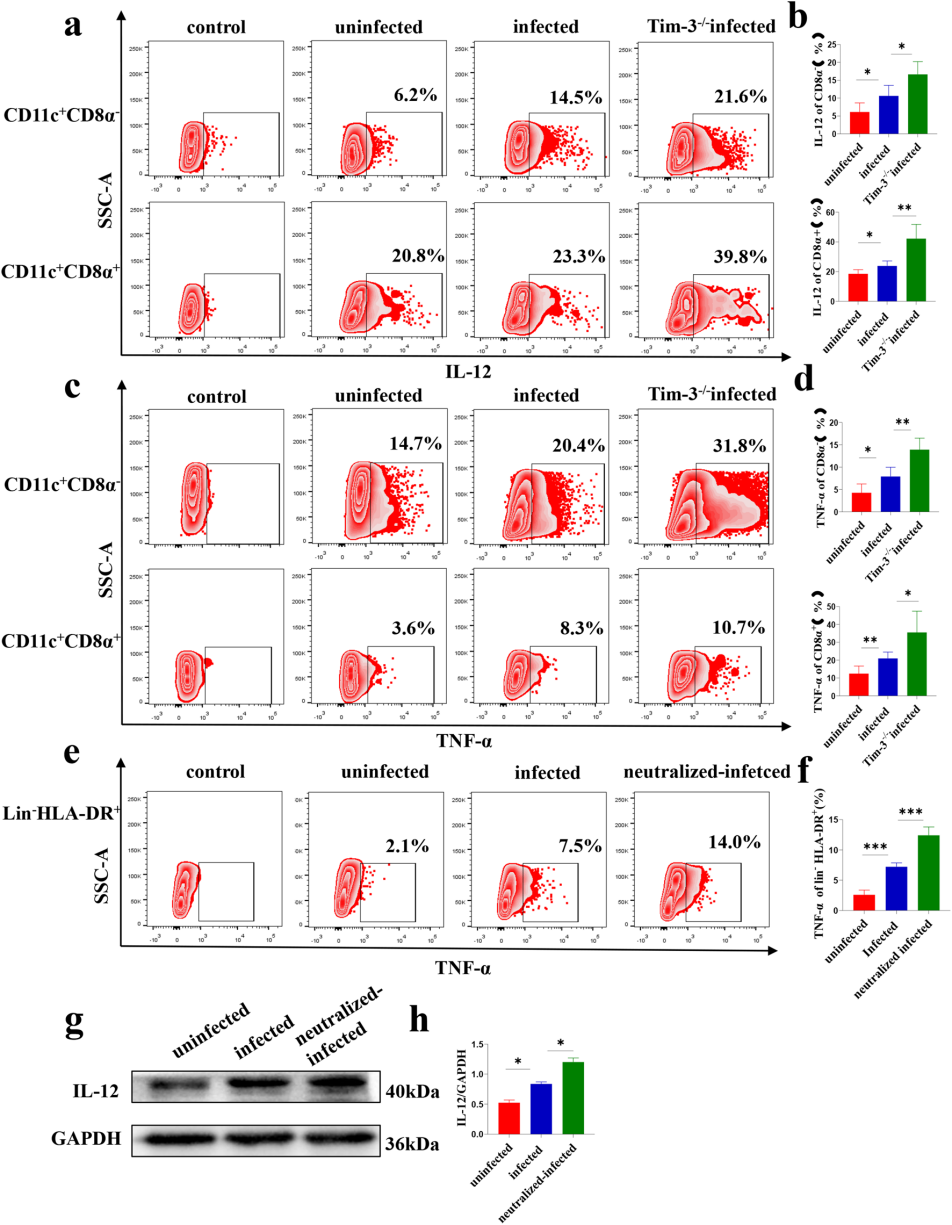
a–i Tim-3 downregulation on human and murine dDCs after *Toxoplasma gondii* infection is associated with the expression of interleukin-12 (*IL-12*) and tumor necrosis factor-α (*TNF-α*). The levels of IL-12 (**a**) and TNF-α (**c**) on myeloid and lymphoid dDCs from uninfected, infected, and infected and Tim-3^−/−^ mice were analyzed by flow cytometry. The levels of IL-12 (**b**) and TNF-α (**d**) on myeloid and lymphoid dDCs from uninfected, infected, and infected and Tim-3^−/−^ mice were statistically analyzed. **e** The expression of TNF-α on human myeloid dDCs from uninfected, infected, and Tim-3-neutralized plus infected groups was analyzed by flow cytometry. **h** The levels of IL-12 on human myeloid dDCs from uninfected, infected and Tim-3-neutralized plus infected groups was analyzed by western blotting. **f**, **i** The expression of TNF-α and IL-12 in human dDCs was statistically analyzed. The data shown are the means ± SD. At least six samples of human decidua in each group were assayed individually by flow cytometry, and at least three samples of human decidua in each group were assayed individually by western blotting. Differences in the results were identified by paired *t*-test. At least six pregnant mice in each group were assayed individually, and differences in the results identified by unpaired *t*-test: uninfected vs. infected group. * P < 0.05, ** P < 0.01, *** P < 0.001

To analyze the effect of Tim-3 downregulation after *T. gondii* infection on cytokines in dDCs, the expression of IL-12 and TNF-α in murine dDCs was analyzed by flow cytometry. TNF-α and IL-12 levels were increased in the infected Tim-3^−/−^ group compared with the infected group [IL-12 including CD11c^+^CD8α^−^, *t*-test, *t*(5) = 3.225, *P* = 0.0233 and CD11c^+^CD8α^+^, *t*-test, *t*(10) = 4.426, *P* = 0.0013] (Fig. 5a, b) [TNF-α including CD11c^+^CD8α^−^, *t*-test, *t*(10) = 4.453, *P* = 0.0012; CD11c^+^CD8α^+^, *t*-test, *t*(10) = 2.856, *P* = 0.0171] (Fig. 5c, d). Additionally, IL-12 and TNF-α levels in Tim-3-neutralized and infected human dDCs were increased compared with those in infected human dDCs, as determined by flow cytometry and western blotting [*t*-test, *t*(2) = 6.291, *P* = 0.0243] [Lin^−^HLA-DR^+^, *t*-test, *t*(5) = 8.777, *P* = 0.0003] (Fig. 5e–h). These results demonstrated that Tim-3 downregulation after *T. gondii* infection increased IL-12 and TNF-α expression.

### *T. gondii* infection-induced Tim-3 downregulation regulated the expression of IL-10 through the SRC-STAT3 signaling pathway

**Fig. 6:**
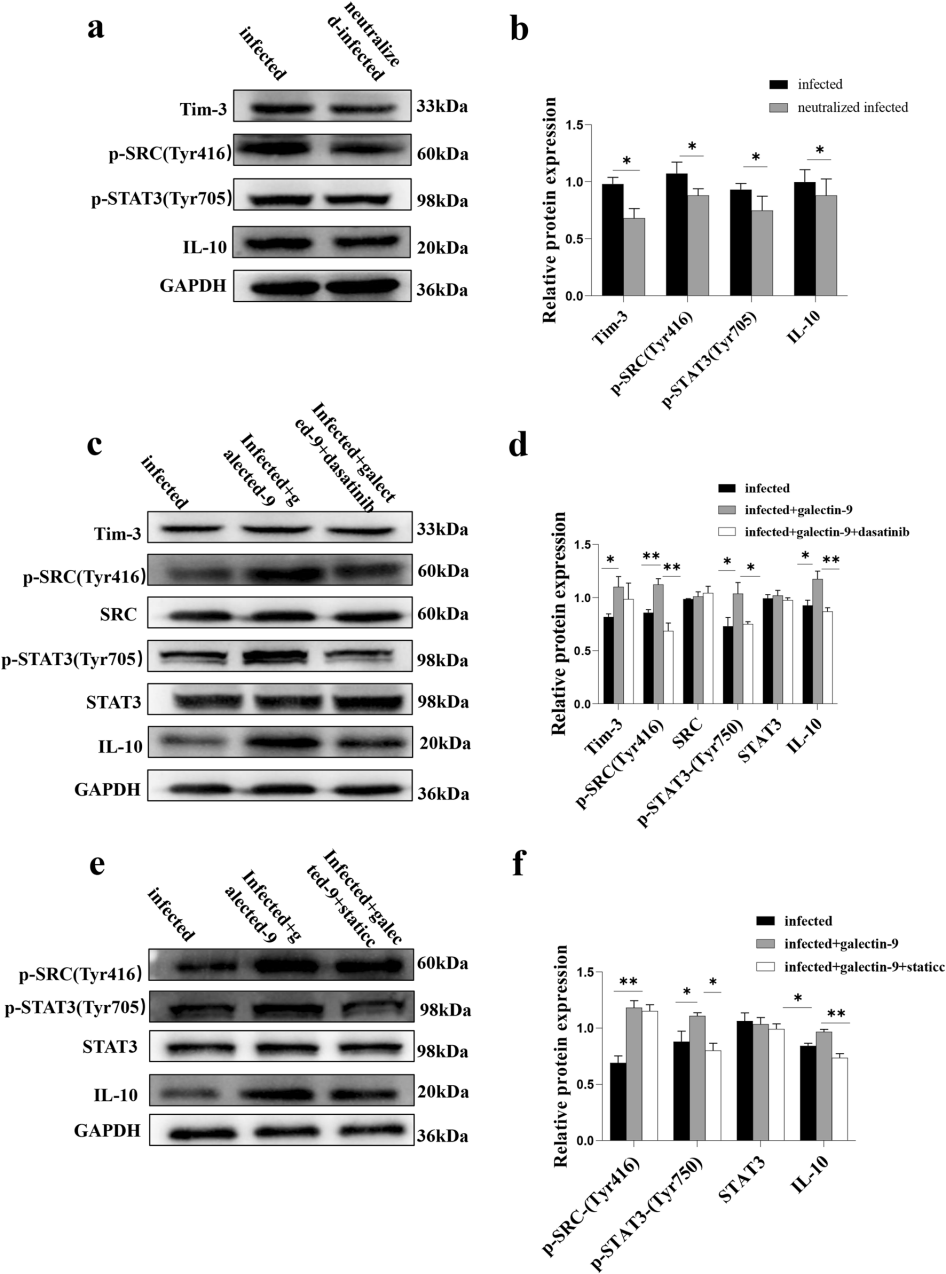
a–f Tim-3 downregulation on dDCs by *Toxoplasma gondii* infection is associated with IL-10 expression via regulation of the SRC-signal transducer and activator of transcription 3 (*STAT3*) pathway. **a**, **b** The levels of Tim-3, p-SRC (Tyr416), p-STAT3 (Tyr705) and IL-10 in purified human dDCs from the infected and Tim-3-neutralized plus infected groups were determined by western blotting and the results examined by statistical analysis. **c**, **d** The levels of Tim-3, p-SRC (Tyr416), SRC, p-STAT3 (Tyr705), STAT3 and IL-10 in purified human dDCs from the infected, infected plus galectin-9 and infected plus galectin-9 plus dasatinib groups were determined by western blotting, and the results examined by statistical analysis. **e**, **f** The levels of Tim-3, p-SRC (Tyr416), SRC, p-STAT3 (Tyr705), STAT3 and IL-10 in purified human dDCs from the infected, infected plus galectin-9 and infected plus galectin-9 plus Stattic groups were determined by western blotting and examined by statistical analysis. The data shown are the means ± SD. At least three samples of human decidua in each group were assayed individually by western blotting, and differences in the results identified by paired *t*-test. * P < 0.05, ** P < 0.01

To observe whether the decrease in tolerant cytokine IL-10 expression in human dDCs resulted from Tim-3 downregulation after *T. gondii* infection, the signaling molecules p-SRC (Tyr416) and p-STAT3 (Tyr705) were measured by western blotting. Levels of p-SRC and p-STAT3 in Tim-3-neutralized and infected human dDCs were lower than in the infected group [p-SRC (Tyr416), *t*-test, *t*(2) = 7.484, *P* = 0.0174; p-STAT3 (Tyr705), *t*-test, *t*(2) = 7.388, *P* = 0.0178] (Fig. 6a, b). These results indicated that Tim-3 downregulation in dDCs during *T. gondii* infection may regulate the expression of IL-10 through the SRC-STAT3 signaling pathway. The molecular mechanism by which Tim-3 is reduced after *T. gondii* infection and leads to the decrease in IL-10 was further elucidated. Galectin-9 (a ligand of Tim-3) was used to activate Tim-3 in infected primary human dDCs. Tim-3, p-SRC, p-STAT3 and IL-10 levels were examined by western blotting. Tim-3, p-SRC, p-STAT3 and IL-10 levels were higher in galectin-9-treated infected dDCs than in infected dDCs [Tim-3, *t*-test, *t*(2) = 5.554, *P* = 0.0309; p-SRC, *t*-test, *t*(2) = 11.56, *P* = 0.0074; p-STAT3, *t*-test, *t*(2) = 5.453, *P* = 0.0320; IL-10, *t*-test, *t*(2) = 6.280, *P* = 0.0244] (Fig. 6c, d).

To further verify whether SRC kinase can phosphorylate STAT3 in response to Tim-3 activation after *T. gondii* infection, dasatinib (a SRC inhibitor) was used to block SRC phosphorylation. The levels of p-SRC, p-STAT3 (Tyr705) and IL-10 were decreased in infected dDCs treated with galectin-9 plus dasatinib compared with infected dDCs treated with galectin-9 alone [p-SRC, *t*-test, *t*(2) = 28.43, *P* = 0.0012; p-STAT3(Tyr705), *t*-test, *t*(2) = 4.359, *P* = 0.0488; IL-10, *t*-test, *t*(2) = 12.23, *P* = 0.0066]. The expression levels of Tim-3, SRC and STAT3 remained unchanged (Fig. 6c, d). These results indicated that the change in Tim-3 expression in dDCs after *T. gondii* infection regulated the expression of IL-10 through SRC phosphorylation.

Next, Stattic (a STAT3 inhibitor) was added to the primary human dDCs during Tim-3 activation. The protein levels of p-STAT3 (Tyr705) and IL-10 in infected dDCs treated with galectin-9 plus Stattic were decreased compared with those in infected dDCs treated with galectin-9 alone [*t*-test, *t*(2) = 7.513, *P* = 0.0173], [*t*-test, *t*(2) = 11.66, *P* = 0.0073]. However, p-SRC (Tyr416) expression and STAT3 expression were not different after the addition of the STAT3 inhibitor. Therefore, our results confirmed that the decrease in Tim-3 in dDCs induced by *T. gondii* infection regulated IL-10 expression through the SRC-STAT3 signaling pathway (Fig. 6e, f).

## Discussion

dDCs, which account for approximately 1–7% of mononuclear cells in the maternal–fetal interface, which normally fail to initiate immunogenic T-cell responses to placental antigens, can help to maintain maternal–fetal tolerance during successful pregnancy [33, 34]. The maternal–fetal tolerance maintained mainly by decidual immune cells depends on several negative regulatory proteins, such as Tim-3, LILRB4 and B7-H4 [16–18]. The studies in our group previously showed that downregulation of LILRB4 on dDCs, especially on the tolerogenic dDC subset, which occurred after *T. gondii* infection, weakened the immune tolerogenic effect of dDCs by upregulating the expression of CD80, CD86 and HLA-DR (MHC-II) and ultimately contributed to abnormal pregnancy outcomes [17]. It has been suggested that Tim-3 in dNK and dMφ plays an important role in adverse pregnancy outcomes caused by *T. gondii* infection [19, 20, 35]. However, it remains unclear whether *T. gondii* infection can change the expression of Tim-3 on dDCs, cause dDC dysfunction and further contribute to *T. gondii*-mediated adverse pregnancy outcomes.

Some studies have reported that Tim-3 is a key mediator that maintains maternal–fetal immunotolerance and successful pregnancy [36, 37]. In a previous study, we demonstrated that abnormal pregnancy outcomes in *T. gondii*-infected and pregnant Tim-3^−/−^ mice were more severe than those in infected and pregnant WT mice; therefore, Tim-3 may play an important role in abnormal pregnancy outcomes induced by *T. gondii* infection [20]. We also showed that the expression levels of Tim-3 on dNK cells and dMφs were decreased after *T. gondii* infection, which damaged maternal–fetal tolerance and eventually led to adverse pregnancy outcomes [19, 20]. In the present study, the expression level of Tim-3 on murine myeloid and lymphoid dDCs was significantly decreased after *T. gondii* infection. Similar changes in Tim-3 expression on human dDCs were observed after infection. These results indicate that Tim-3 downregulation on dDCs after *T. gondii* infection can disrupt the balance of maternal–fetal tolerance and may promote the process of adverse pregnancy outcomes.

One study showed that blocking CD86 in abortion-prone mouse models could promote maternal–fetal immune tolerance, thereby improving pregnancy outcomes [38]. Another that DCs derived from the peripheral blood of preeclampsia patients expressed increased levels of CD80 and CD86 [39]. The absence of MHC-II molecules from trophoblast layers appears to be an important feature for fetal survival [40]. In previous work we showed that the decreased expression of LILRB4 and B7-H4 on dDCs induced by *T. gondii* infection could increase the expression of CD80, CD86 and MHC-II, leading to dDC dysfunction and contributing to abnormal pregnancy outcomes [17, 41]. To determine whether the decreased expression of Tim-3 after *T. gondii* infection could lead to changes in CD80, CD86 and MHC-II expression on dDCs, infected and pregnant Tim-3^−/−^ mice with adverse pregnancy outcomes were used in the present study. The expression levels of CD80, CD86 and MHC-II on dDCs in infected Tim-3^−/−^ mice were increased compared with those in infected WT mice. The expression of CD86 on infected human dDCs was upregulated after treatment with Tim-3 neutralizing antibodies. These results indicated that Tim-3 downregulation on dDCs after *T. gondii* infection could increase CD80, CD86 and MHC-II expression, therefore impairing the maternal–fetal tolerance function of dDCs.

IDO is an enzyme that participates in the catabolism of tryptophan, which is essential for T-cell proliferation [42, 43]. The metabolite kynurenine is involved in the inhibition of T and NK cells and the generation of Treg cells, and directly regulates the immune response by educating the immune microenvironment in vivo [44, 45]. IDO plays an important role in normal pregnancy through immune suppression and the regulation of fetal invasion [46]. A report showed that, after CTLA-4 treatment, IDO expression on dDCs was increased in normal pregnancy but that it was decreased in cases of spontaneous abortion [47], suggesting the vital role of IDO on dDCs in the maintenance of pregnancy. Other studies have shown that abnormal expression of IDO is related to the occurrence of some pathological pregnancies, such as recurrent spontaneous abortion, preeclampsia, premature delivery and intrauterine growth retardation [46, 48, 49]. Our recent research also clarified that the reduction in B7-H4 by *T. gondii* infection gave rise to a decrease in IDO expression in dDCs [41]. However, whether the decrease in Tim-3 on dDCs after *T. gondii* infection can affect IDO synthesis in dDCs is still unclear. In *T. gondii*-infected Tim-3^−/−^ mice, the IDO expression level was decreased compared with that in infected WT mice. Furthermore, IDO expression on *T. gondii*-infected human dDCs was also reduced after blocking Tim-3 with a Tim-3 neutralizing antibody. These results suggested that the Tim-3 downregulation after *T. gondii* infection reduced IDO synthesis and disordered dDC-mediated maternal–fetal tolerance function.

IL-10 plays beneficial roles in normal pregnancy as one of the key cytokines in the Th2-type response [50]. IL-10 plays an immune protective role and was reported to improve the pregnancy outcome of *T. gondii*-infected mice [51]. Studies have shown a significant reduction in placental IL-10 mRNA and protein expression in women with preeclampsia compared to controls [52]. Similar to previous studies in our group which showed that the Tim-3 downregulation on dNK cells and dMφs caused by *T. gondii* infection could lead to a decrease in IL-10 expression [19, 20], our results proved that, in dDCs, Tim-3 downregulation after *T. gondii* infection also could decrease the synthesis of IL-10 and further result in dDC dysfunction.

Conversely, IL-12 is considered to be a major proinflammatory cytokine that can stimulate the synthesis of nitric oxide and other mediators of inflammation [53, 54]. Our recent study showed that expression of IL-12 in murine and primary human dDCs was increased after *T. gondii* infection [41]. TNF-α is also a major inflammatory cytokine that promotes various inflammatory responses [55]. A study demonstrated that certain pathological pregnancies, such as recurrent abortion, premature delivery and severe preeclampsia, as well as recurrent implantation failure syndrome, are closely associated with elevated Th1 cytokines, especially TNF-α [56]. Tim-3 has been showed to negatively regulate IL-12 expression on monocytes during hepatitis C virus infection [27]. Blocking the Tim-3 signaling pathway significantly increased TNF-α levels in the supernatant of T lymphocytes of patients with sepsis [57]. However, whether the decrease of Tim-3 induced by *T. gondii* infection could regulate the secretion of IL-12 and TNF-α by dDCs still needs further examination. Therefore, we analyzed the expression levels of IL-12 and TNF-α in infected Tim-3^−/−^ mice and infected human dDCs that were treated with Tim-3 neutralizing antibodies. The results showed that the expression levels of IL-12 and TNF-α were further increased in infected Tim-3^−/−^ mice and in infected human dDCs that were treated with Tim-3 neutralizing antibodies compared to the infected pregnant mice or infected human dDcs, respectively. Our results demonstrated that the decrease of Tim-3 after *T. gondii* infection could upregulate IL-12 and TNF-α expression, which may weaken the maternal–fetal tolerance function of dDCs.

In a lipopolysaccharide-treated preeclampsia model, Tim-3 activation induced by galectin-9 could significantly upregulate IL-10 mRNA levels in dMφs [8]. Tim-3 pathway blockade resulted in reduced IL-10 production in decidual immune cells [16]. Our results proved that Tim-3 downregulation after *T. gondii* infection decreased IL-10 expression in dDCs. However, the detailed molecular mechanism by which Tim-3 regulates IL-10 expression in dDCs during *T. gondii* infection is still unclear. Tim-3 has been reported to inhibit DC activation through Bruton’s tyrosine kinase-SRC pathway [30, 31]. Activated SRC kinase can directly phosphorylate STAT3; subsequently, dimerized p-STAT3 translocates to the nucleus, where it binds to the promoter regions of IL-10 [32]. To examine the molecular mechanism by which Tim-3 regulates IL-10 expression in dDCs after *T. gondii* infection, the expression levels of p-SRC (Tyr416), p-STAT3 (Tyr705) and IL-10 in Tim-3-neutralized and infected human dDCs were examined by western blotting. p-SRC (Tyr416), p-STAT3 (Tyr705) and IL-10 expression were lower in Tim-3-neutralized and infected dDCs than in infected dDCs. To further examine the signaling pathway by which IL-10 is reduced by the decrease in Tim-3 after *T. gondii* infection, galectin-9 (a ligand of Tim-3) was used to activate Tim-3 in purified human dDCs, and dasatinib and Stattic were used to block SRC and STAT3 kinase activity, respectively. The results showed that p-SRC, p-STAT3 and IL-10 were increased in infected human dDCs after galectin-9 was used to activate Tim-3, while the expression of p-STAT3 and IL-10 were decreased when dasatinib was used to block SRC kinase activity, and IL-10 was reduced by static blockade of STAT3 phosphorylation. These results suggested that the reduction in Tim-3 in *T. gondii*-infected dDCs could decrease the phosphorylation of SRC and STAT3, ultimately regulating IL-10 expression through the SRC-STAT3 signaling pathway.

## Conclusions

In summary, *T. gondii* infection significantly downregulated Tim-3 expression on dDCs. The reduction in Tim-3 could increase functional molecules (CD80, CD86, MHC-II) and cytokines (TNF-α and IL-12) expression and decrease IDO synthesis and IL-10 expression. Moreover, the decrease in Tim-3 after *T. gondii* infection ultimately regulated IL-10 expression through the SRC-STAT3 signaling pathway. The changes in these functional molecules induced by Tim-3 downregulation during *T. gondii* infection led to dDC dysfunction, which may have contributed to the adverse pregnancy outcomes. This study provides further new insights into the immune molecular mechanism of abnormal pregnancy outcomes mediated by *T. gondii* infection.

## Supplementary Information

**Additional file 1:** **Table S1. **Antibodies (catalogue no., vendor, clone, fluorochrome, concentration) used in this study.

**Additional file 2: Figure S1.** Flow cytometry gating strategy for dDC.** a** In humans, the P1 gate was based on forward and side scatter (FSC-A and SSC-A) to remove dead cells and cell fragments. Then, myeloid DCs in P2 (lineage^-^HLA-DR^+^) were gated out using the markers Lineage and HLA-DR. **b** For pregnant female mice, P1 representative dot plots were gated on forward versus side scatter (FSC/SSC) to remove dead cells and cell fragments. P2 CD11c^+^ cells were gated on selected monocytes. P4 CD8α^+^ cells were selected among CD11c^+^ cells to show the percentage of lymphatic DCs, and P3 CD8α^-^ cells were selected among CD11^+^ cells to show the percentage of myeloid DCs.

## Data Availability

All the data generated in this study are presented within the published article.

## Abbreviations

B7-H4: B7 homolog 4
DC: Dendritic cell
dDC: Decidual dendritic cell
GD: Gestational day
H&E: Hematoxylin and eosin
IDO: Indoleamine 2,3-dioxygenase
IL-10: Interleukin-10
IL-12: Interleukin-12
LILRB4: Leukocyte immunoglobulin-like receptor B4
Mφ: Macrophage
NK: Natural killer
PBS: Phosphate buffered saline
p-STAT3: Phosphorylated STAT3
STAT3: Signal transducer and activator of transcription 3
TIM-3: T cell immunoglobulin and mucin domain 3
Tim-3^−/−^: T cell immunoglobulin and mucin domain 3 deficient
TNF-α: Tumor necrosis factor-α
WT: Wild type

## References

[1] Tenter AM, Heckeroth AR, Weiss LM (2000). *Toxoplasma gondii*: from animals to humans. Int J Parasitol.

[2] Gazzonis AL, Veronesi F, Di Cerbo AR, Zanzani SA, Molineri G, Moretta I (2015). *Toxoplasma gondii* in small ruminants in northern Italy-prevalence and risk factors. Ann Agric Environ Med.

[3] Arora N, Sadovsky Y, Dermody TS, Coyne CB (2017). Microbial vertical transmission during human pregnancy. Cell Host Microbe.

[4] Montoya JG, Remington JS (2008). Management of *Toxoplasma gondii* infection during pregnancy. Clin Infect Dis.

[5] Hunt JS, Robertson SA (1996). Uterine macrophages and environmental programming for pregnancy success. J Reprod Immunol.

[6] Zhou Y, Fu B, Xu X, Zhang J, Tong X, Wang Y (2020). PBX1 expression in uterine natural killer cells drives fetal growth. Sci Transl Med.

[7] Chen P, Zhou L, Chen J, Lu Y, Cao C, Lv S (2021). The immune atlas of human deciduas with unexplained recurrent pregnancy loss. Front Immunol.

[8] Li ZH, Wang LL, Liu H, Muyayalo KP, Huang XB, Mor G (2019). Galectin-9 alleviates LPS-induced preeclampsia-like impairment in rats via switching decidual macrophage polarization to M2 subtype. Front Immunol.

[9] Moffett A, Chazara O, Colucci F, Johnson MH (2016). Variation of maternal KIR and fetal HLA-C genes in reproductive failure: too early for clinical intervention. Reprod Biomed Online.

[10] Kämmerer U, Eggert AO, Kapp M, McLellan AD, Geijtenbeek TB, Dietl J (2003). Unique appearance of proliferating antigen-presenting cells expressing DC-SIGN (CD209) in the decidua of early human pregnancy. Am J Pathol.

[11] Miyazaki S, Tsuda H, Sakai M, Hori S, Sasaki Y, Futatani T (2003). Predominance of Th2-promoting dendritic cells in early human pregnancy decidua. J Leukoc Biol.

[12] Juretic K, Strbo N, Crncic TB, Laskarin G, Rukavina D (2004). An insight into the dendritic cells at the maternal-fetal interface. Am J Reprod Immunol.

[13] Banchereau J, Briere F, Caux C, Davoust J, Lebecque S, Liu YJ (2000). Immunobiology of dendritic cells. Annu Rev Immunol.

[14] Ahmadabad HN, Salehnia M, Saito S, Moazzeni SM (2016). Decidual soluble factors, through modulation of dendritic cells functions, determine the immune response patterns at the feto-maternal interface. J Reprod Immunol.

[15] Tirado-González I, Muñoz-Fernández R, Blanco O, Leno-Durán E, Abadía-Molina AC, Olivares EG (2010). Reduced proportion of decidual DC-SIGN+ cells in human spontaneous abortion. Placenta.

[16] Wang S, Chen C, Sun F, Li M, Du M, Li X (2021). Involvement of the Tim-3 pathway in the pathogenesis of pre-eclampsia. Reprod Sci.

[17] Zhan S, Zheng J, Zhang H, Zhao M, Liu X, Jiang Y (2018). LILRB4 decrease on uDCs exacerbate abnormal pregnancy outcomes following *Toxoplasma gondii* infection. Front Microbiol.

[18] Darmochwal-Kolarz D, Kludka-Sternik M, Kolarz B, Chmielewski T, Tabarkiewicz J, Rolinski J (2013). The expression of B7–H1 and B7–H4 co-stimulatory molecules on myeloid and plasmacytoid dendritic cells in pre-eclampsia and normal pregnancy. J Reprod Immunol.

[19] Li T, Cui L, Xu X, Zhang H, Jiang Y, Ren L (2021). The role of Tim-3 on dNK cells dysfunction during abnormal pregnancy With *Toxoplasma gondii* infection. Front Cell Infect Microbiol.

[20] Zhang D, Ren L, Zhao M, Yang C, Liu X, Zhang H (2019). Role of Tim-3 in decidual macrophage functional polarization during abnormal pregnancy with *Toxoplasma gondii* infection. Front Immunol.

[21] Li Z, Zhao M, Li T, Zheng J, Liu X, Jiang Y (2017). Decidual macrophage functional polarization during abnormal pregnancy due to *Toxoplasma gondii*: role for LILRB4. Front Immunol.

[22] Zhu C, Anderson AC, Schubart A, Xiong H, Imitola J, Khoury SJ (2005). The Tim-3 ligand galectin-9 negatively regulates T helper type 1 immunity. Nat Immunol.

[23] Gupta S, Thornley TB, Gao W, Larocca R, Turka LA, Kuchroo VK (2012). Allograft rejection is restrained by short-lived TIM-3+PD-1+Foxp3+ Tregs. J Clin Invest.

[24] Gleason MK, Lenvik TR, McCullar V, Felices M, O'Brien MS, Cooley SA (2012). Tim-3 is an inducible human natural killer cell receptor that enhances interferon gamma production in response to galectin-9. Blood.

[25] Chiba S, Baghdadi M, Akiba H, Yoshiyama H, Kinoshita I, Dosaka-Akita H (2012). Tumor-infiltrating DCs suppress nucleic acid-mediated innate immune responses through interactions between the receptor TIM-3 and the alarmin HMGB1. Nat Immunol.

[26] Wang S, Chen C, Li M, Qian J, Sun F, Li Y (2019). Blockade of CTLA-4 and Tim-3 pathways induces fetal loss with altered cytokine profiles by decidual CD4(+)T cells. Cell Death Dis.

[27] Zhang Y, Ma CJ, Wang JM, Ji XJ, Wu XY, Jia ZS (2011). Tim-3 negatively regulates IL-12 expression by monocytes in HCV infection. PLoS ONE.

[28] Li YH, Zhou WH, Tao Y, Wang SC, Jiang YL, Zhang D (2016). The galectin-9/Tim-3 pathway is involved in the regulation of NK cell function at the maternal-fetal interface in early pregnancy. Cell Mol Immunol.

[29] Azizieh FY, Raghupathy R (2017). IL-10 and pregnancy complications. Clin Exp Obstet Gynecol.

[30] Maurya N, Gujar R, Gupta M, Yadav V, Verma S, Sen P (2014). Immunoregulation of dendritic cells by the receptor T cell Ig and mucin protein-3 via Bruton’s tyrosine kinase and c-Src. J Immunol.

[31] Norkina O, Dolganiuc A, Shapiro T, Kodys K, Mandrekar P, Szabo G (2007). Acute alcohol activates STAT3, AP-1, and Sp-1 transcription factors via the family of Src kinases to promote IL-10 production in human monocytes. J Leukoc Biol.

[32] Zhang X, Blenis J, Li HC, Schindler C, Chen-Kiang S (1995). Requirement of serine phosphorylation for formation of STAT-promoter complexes. Science.

[33] Monin L, Whettlock EM, Male V (2020). Immune responses in the human female reproductive tract. Immunology.

[34] Erlebacher A (2013). Immunology of the maternal-fetal interface. Annu Rev Immunol.

[35] Fu X, Wu B, Huang B, Zheng H, Huang S, Gan Y (2015). The correlation of Tim-3 and IFN-gamma expressions in mice infected with *Toxoplasma gondii* during gestation. Parasitol Res.

[36] Wang SC, Li YH, Piao HL, Hong XW, Zhang D, Xu YY (2015). PD-1 and Tim-3 pathways are associated with regulatory CD8+ T-cell function in decidua and maintenance of normal pregnancy. Cell Death Dis.

[37] Meggyes M, Lajko A, Palkovics T, Totsimon A, Illes Z, Szereday L (2015). Feto-maternal immune regulation by TIM-3/galectin-9 pathway and PD-1 molecule in mice at day 14.5 of pregnancy. Placenta.

[38] Zhu XY, Zhou YH, Wang MY, Jin LP, Yuan MM, Li DJ (2005). Blockade of CD86 signaling facilitates a Th2 bias at the maternal-fetal interface and expands peripheral CD4+CD25+ regulatory T cells to rescue abortion-prone fetuses. Biol Reprod.

[39] Wang J, Tao YM, Cheng XY, Zhu TF, Chen ZF, Yao H (2014). Dendritic cells derived from preeclampsia patients influence Th1/Th17 cell differentiation in vitro. Int J Clin Exp Med.

[40] Vassiliadis S, Tsoukatos D, Athanassakis I (1994). Interferon-induced class II expression at the spongiotrophoblastic zone of the murine placenta is linked to fetal rejection and developmental abnormalities. Acta Physiol Scand.

[41] Sun X, Xie H, Zhang H, Li Z, Qi H, Yang C (2022). B7–H4 reduction induced by *Toxoplasma gondii* infection results in dysfunction of decidual dendritic cells by regulating the JAK2/STAT3 pathway. Parasit Vectors.

[42] Sedlmayr P, Blaschitz A, Wintersteiger R, Semlitsch M, Hammer A, MacKenzie CR (2002). Localization of indoleamine 2,3-dioxygenase in human female reproductive organs and the placenta. Mol Hum Reprod.

[43] Yuasa HJ, Takubo M, Takahashi A, Hasegawa T, Noma H, Suzuki T (2007). Evolution of vertebrate indoleamine 2,3-dioxygenases. J Mol Evol.

[44] Yu LL, Zhang YH, Zhao FX (2017). Expression of indoleamine 2,3-dioxygenase in pregnant mice correlates with CD4+CD25+Foxp3+ T regulatory cells. Eur Rev Med Pharmacol Sci.

[45] Munn DH, Mellor AL (2013). Indoleamine 2,3 dioxygenase and metabolic control of immune responses. Trends Immunol.

[46] Chang RQ, Li DJ, Li MQ (2018). The role of indoleamine-2,3-dioxygenase in normal and pathological pregnancies. Am J Reprod Immunol.

[47] Miwa N, Hayakawa S, Miyazaki S, Myojo S, Sasaki Y, Sakai M (2005). IDO expression on decidual and peripheral blood dendritic cells and monocytes/macrophages after treatment with CTLA-4 or interferon-gamma increase in normal pregnancy but decrease in spontaneous abortion. Mol Hum Reprod.

[48] Ban Y, Chang Y, Dong B, Kong B, Qu X (2013). Indoleamine 2,3-dioxygenase levels at the normal and recurrent spontaneous abortion fetal-maternal interface. J Int Med Res.

[49] Zardoya-Laguardia P, Blaschitz A, Hirschmugl B, Lang I, Herzog SA, Nikitina L (2018). Endothelial indoleamine 2,3-dioxygenase-1 regulates the placental vascular tone and is deficient in intrauterine growth restriction and pre-eclampsia. Sci Rep.

[50] Cubro H, Kashyap S, Nath MC, Ackerman AW, Garovic VD (2018). The role of interleukin-10 in the pathophysiology of preeclampsia. Curr Hypertens Rep.

[51] Zhang R, Zhang H, Liu X, Fu Q, Xu X, Hu X (2012). The immunoprotective role of interleukin-10 in abnormal pregnancy outcome induced by *Toxoplasma gondii* infection. Gynecol Obstet Invest.

[52] Makris A, Xu B, Yu B, Thornton C, Hennessy A (2006). Placental deficiency of interleukin-10 (IL-10) in preeclampsia and its relationship to an IL10 promoter polymorphism. Placenta.

[53] Trinchieri G (1995). Interleukin-12: a proinflammatory cytokine with immunoregulatory functions that bridge innate resistance and antigen-specific adaptive immunity. Annu Rev Immunol.

[54] Trinchieri G (2003). Interleukin-12 and the regulation of innate resistance and adaptive immunity. Nat Rev Immunol.

[55] Menges M, Rossner S, Voigtlander C, Schindler H, Kukutsch NA, Bogdan C (2002). Repetitive injections of dendritic cells matured with tumor necrosis factor alpha induce antigen-specific protection of mice from autoimmunity. J Exp Med.

[56] Saito S, Nakashima A, Shima T, Ito M (2010). Th1/Th2/Th17 and regulatory T-cell paradigm in pregnancy. Am J Reprod Immunol.

[57] Xia Q, Wei L, Zhang Y, Sheng J, Wu W, Zhang Y (2018). Immune checkpoint receptors Tim-3 and PD-1 regulate monocyte and T lymphocyte function in septic patients. Mediators Inflamm.

