# Supplementary figures and images for: Tim-3 downregulation by Toxoplasma gondii infection contributes to decidual dendritic cell dysfunction
Source: Parasit Vectors. 2022 Oct 27;15:393. doi: 10.1186/s13071-022-05506-1 (PMC9615254; doi:10.1186/s13071-022-05506-1)

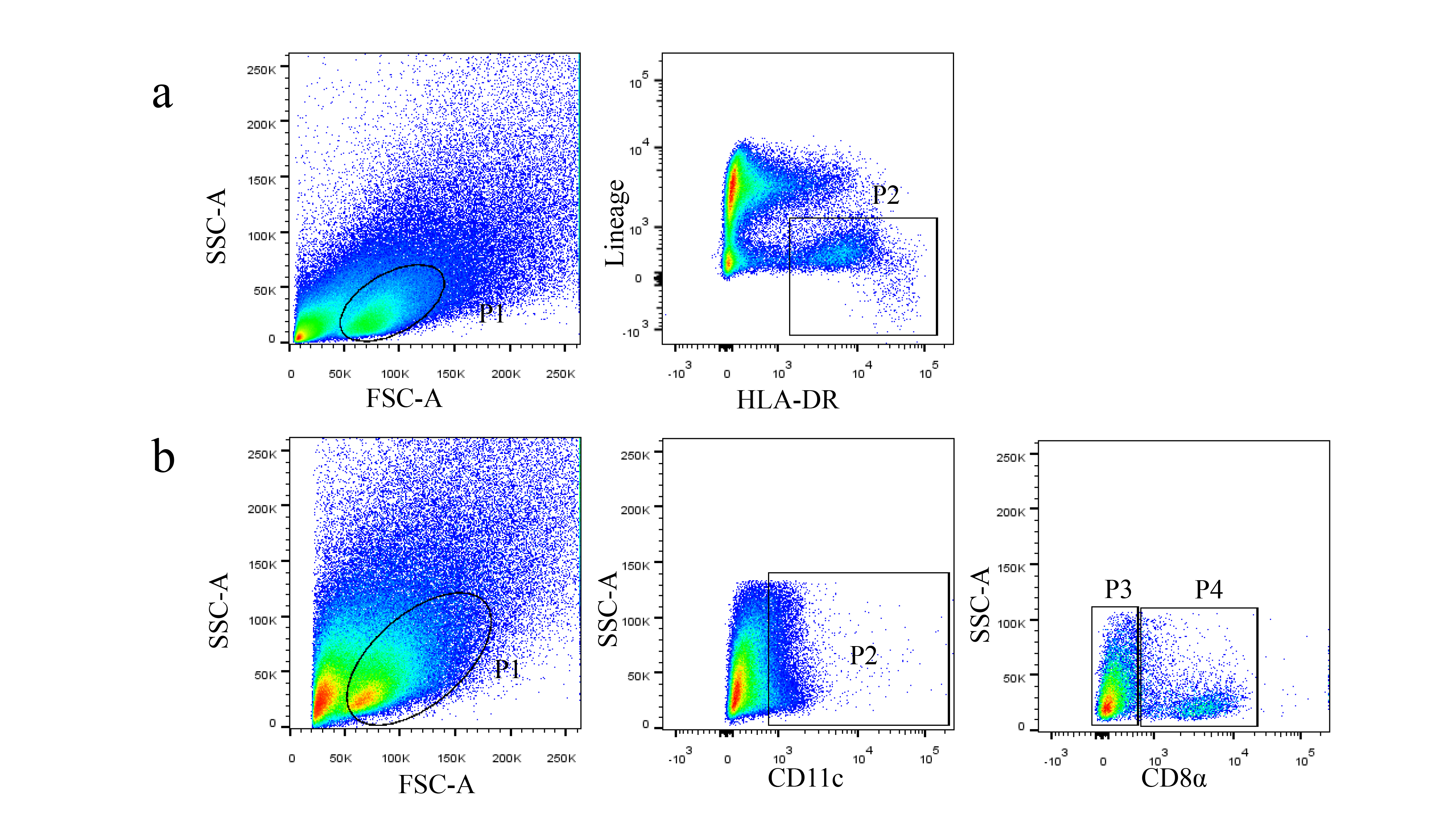

Supplement: Supplementary file 2 — Additional file 2: Figure S1. Flow cytometry gating strategy for dDC. a In humans, the P1 gate was based on forward and side scatter (FSC-A and SSC-A) to remove dead cells and cell fragments. Then, myeloid DCs in P2 (lineage-HLA-DR+) were gated out using the markers Lineage and HLA-DR. b For pregnant female mice, P1 representative dot plots were gated on forward versus side scatter (FSC/SSC) to remove dead cells and cell fragments. P2 CD11c+ cells were gated on selected monocytes. P4 CD8α+ cells were selected among CD11c+ cells to show the percentage of lymphatic DCs, and P3 CD8α- cells were selected among CD11+ cells to show the percentage of myeloid DCs. [file 13071_2022_5506_MOESM2_ESM.tif]
